# Maxillofacial Changes in Melnick-Needles Syndrome

**DOI:** 10.1155/2016/9685429

**Published:** 2016-07-12

**Authors:** Leilane Larissa Albuquerque do Nascimento, Monica da Consolação Canuto Salgueiro, Mariana Quintela, Victor Perez Teixeira, Ana Carolina Costa Mota, Camila Haddad Leal de Godoy, Sandra Kalil Bussadori

**Affiliations:** ^1^Brazilian Association of Dentistry, 57037-240 Maceió, AL, Brazil; ^2^Postgraduate Program in Biophotonics Applied to Health Sciences, Nove de Julho University, 01504-001 São Paulo, SP, Brazil; ^3^Nove de Julho University, 01504-001 São Paulo, SP, Brazil; ^4^Postgraduate Program in Rehabilitation Sciences, Nove de Julho University, 01504-001 São Paulo, SP, Brazil

## Abstract

*Background*. Melnick-Needles Syndrome is rare congenital hereditary skeletal dysplasia caused by mutations in the FLNA gene, which codifies the protein filamin A. This condition leads to serious skeletal abnormalities, including the stomatognathic region.* Case Presentation*. This paper describes the case of a 13-year-old girl diagnosed with Melnick-Needles Syndrome presenting with different forms of skeletal dysplasia, such as cranial hyperostosis, short upper limbs, bowed long bones, metaphyseal thickening, genu valgum (knock-knee), shortened distal phalanges, narrow pelvis and shoulders, rib tapering and irregularities, elongation of the vertebrae, kyphoscoliosis, micrognathia, hypoplastic coronoid processes of the mandible, left stylohyoid ligament suggesting ossification, and dental development anomalies.* Conclusion*. Knowledge of this rare syndrome on the part of dentists is important due to the fact that this condition involves severe abnormalities of the stomatognathic system that cause an impact on the development of the entire face as well as functional and esthetic impairments.

## 1. Introduction

Melnick-Needles Syndrome (MNS; OMIM 309350) is rare congenital hereditary skeletal dysplasia first reported in 1966 by Melnick and Needles. The diagnosis was based on clinical and radiographic characteristics found in two families with some degree of bone involvement in common [1, 2].

This syndrome has an inherited pattern linked to the dominant X, in which there is deficient osteoblastic activity, resulting in characteristic clinical and radiographic findings [2, 3]. The majority of cases reported in the literature involve the female sex, because the clinical form is more severe in males, which leads to miscarriage, fetal death, or neonatal death when born from mothers with this syndrome [4, 5].

MNS is one of the four syndromes caused by mutations in the FLNA gene, which codifies the protein filamin A (Table 1); this protein is related to collagen production [3, 6, 7]. Specifically, MNS is related to a mutation in exon 22 [8]. The other syndromes are frontometaphyseal dysplasia, otopalatodigital syndrome type 1 (OPD1), and otopalatodigital syndrome type 2 (OPD2). Due to different chromosome loci, the phenotypes of these conditions differ from each other and may or may not appear in a severe form [6, 9, 10].

The main clinical and radiographic characteristics of MNS are displayed as follows [1, 10–17].

*Chart of the Main Clinical and Radiographic Characteristics of Melnick-Needles Syndrome*

- Being lethal when affecting sons of mothers with MNS,
- Skeletal dysplasia,
- No palate involvement,
- Shortened distal phalanges,
- Short upper limbs,
- Bowing of long bones,
- Genu valgum,
- Exophthalmia,
- Micrognathia,
- Rib irregularities, elongation of vertebrae, and scoliosis.

The differential diagnosis should include Frank-Ter Haar Syndrome, which is an autosomal recessive disorder that has similar characteristics to MNS but includes congenital glaucoma and heart anomalies [4, 14, 18]. Some radiographic characteristics can be similar to those found in Shprintzen-Goldberg Syndrome, which differs clinically from MNS by the presence of craniosynostosis and mental retardation [9, 19]. The Pierre Robin Sequence, Treacher Collins Syndrome, and Crouzon Syndrome [20–22] should also be parts of the differential diagnosis (Table 2).

## 2. Case Presentation

A 13-year-old female patient, who was previously diagnosed with Melnick-Needles Syndrome (OMIN 309350) at eight years of age, sought dental follow-up at the Specialized Pediatric Dentistry Clinic of the Brazilian Association of Dentistry in the state of Alagoas, Brazil, accompanied by her mother.

### 2.1. Medical Findings

**Figure 1:**
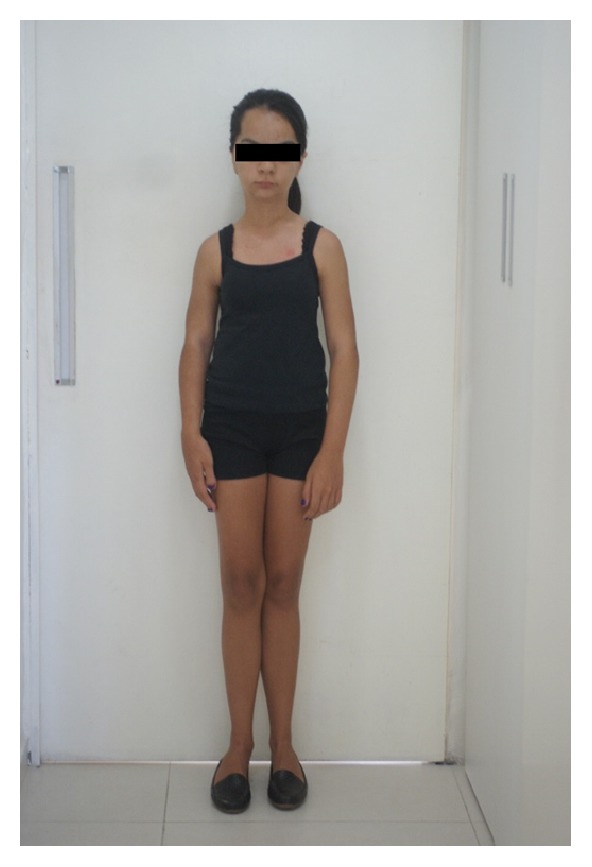
Presence of bowing of long bones, narrow shoulders, prominent brow, micrognathia, and her head being larger than normal.

**Figure 2:**
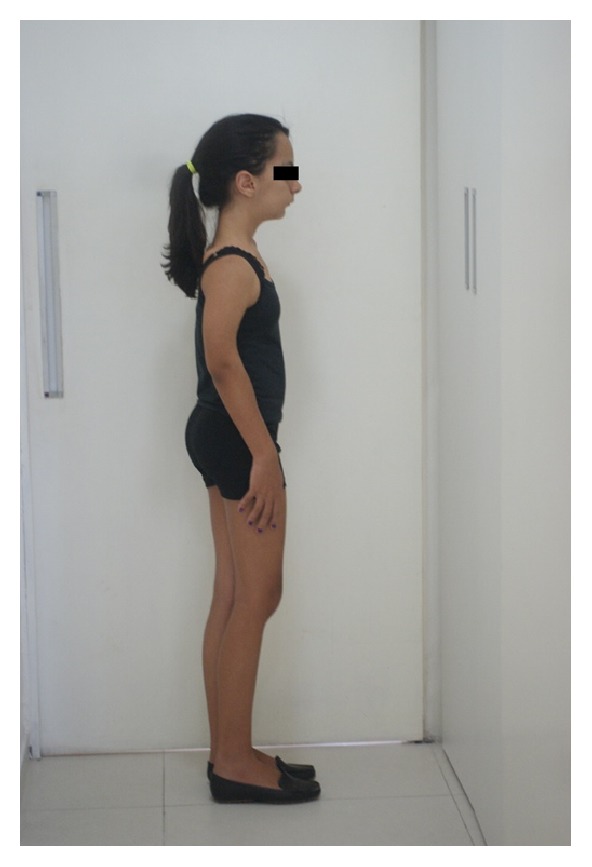
Presence of genu valgum (knock-knee), bowing of long bones, scoliosis, prominent brow, micrognathia, and her head being larger than normal.

**Figure 3:**
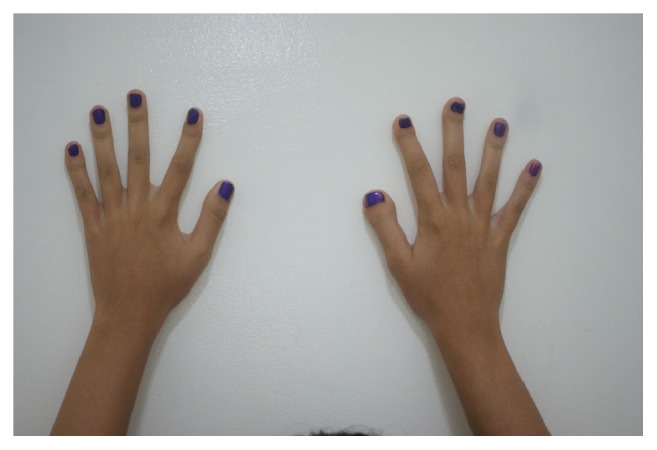
Shortened distal phalanges.

The diagnosis was based on pathognomonic and radiographic findings: small face with a prominent brow, exophthalmia, micrognathia, cranial hyperostosis, poor alignment of the teeth, short upper limbs, bowed long bones (especially the humerus and tibias), metaphyseal thickening, genu valgum, shortened distal phalanges, narrow pelvis and shoulders, tapered ribs with irregularities, elongation of the vertebrae, and kyphoscoliosis (Figures 1, 2, and 3).

### 2.2. Extraoral and Intraoral Findings

**Figure 4:**
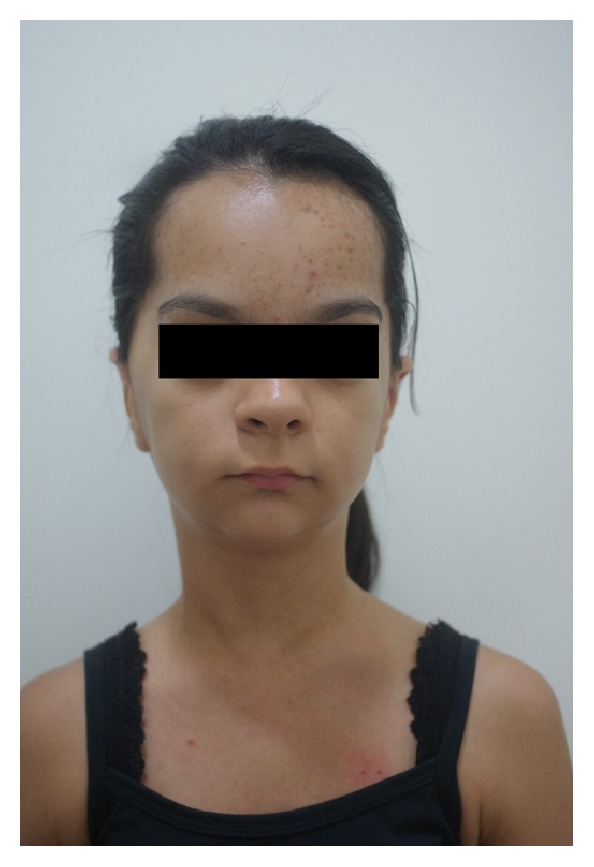
Extraoral photograph of the front: prominent brow and micrognathia.

**Figure 5:**
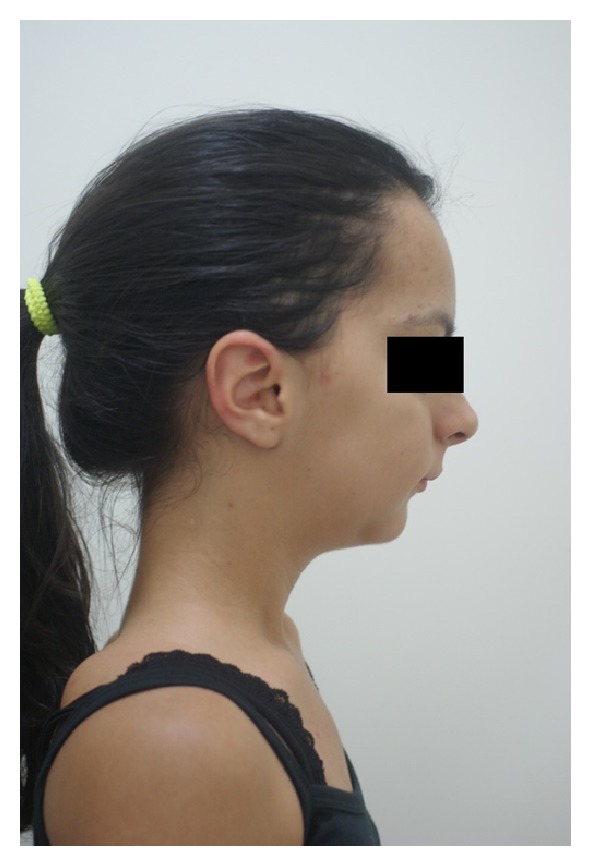
Extraoral photograph, profile: prominent brow and micrognathia.

The extraoral photographs of the face revealed the prominent brow, exophthalmia, and micrognathia (Figures 4 and 5).

**Figure 6:**
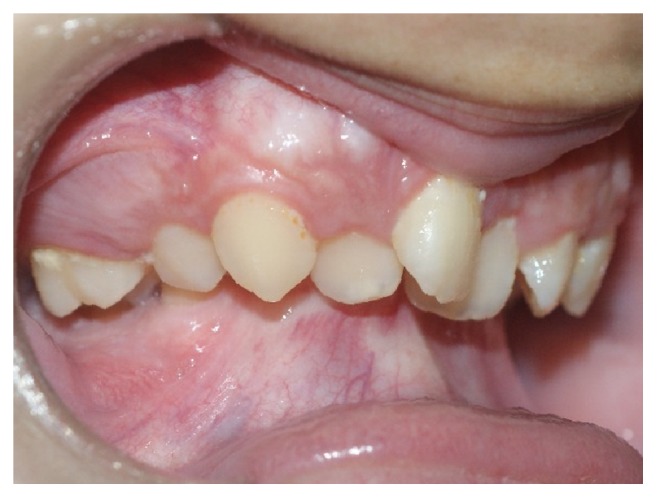
Intraoral photographs, right side: anterior deep bite, maxillary crowding, and accentuated overjet.

**Figure 7:**
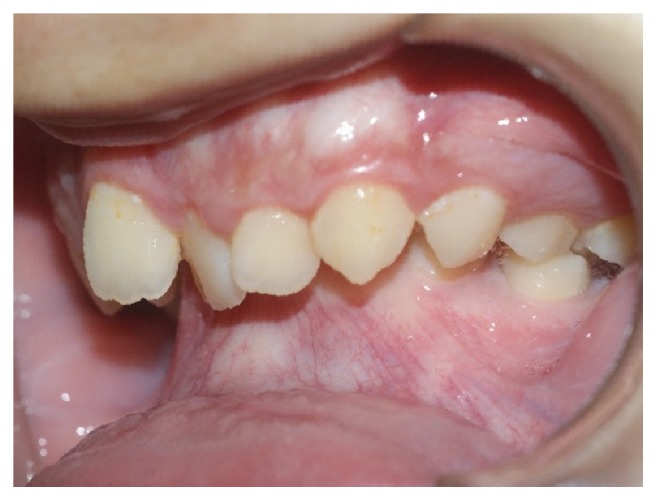
Intraoral photographs, left side: anterior deep bite, maxillary crowding, and accentuated overjet.

**Figure 8:**
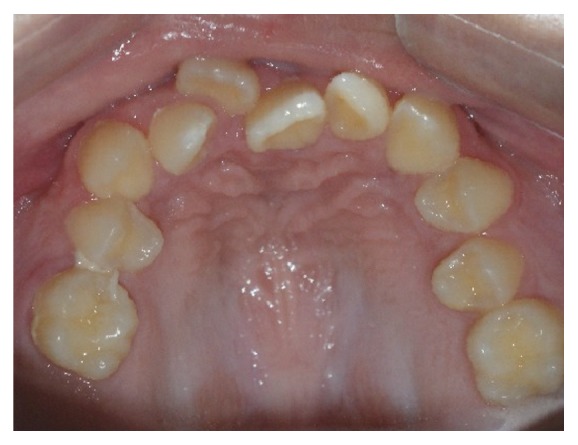
Maxillary occlusal photograph: maxillary crowding and high palate.

Intraoral examination revealed considerably poor tooth alignment, with upper dental crowding and anterior deep bite (Figures 6, 7, and 8).

### 2.3. Radiographic Findings

**Figure 9:**
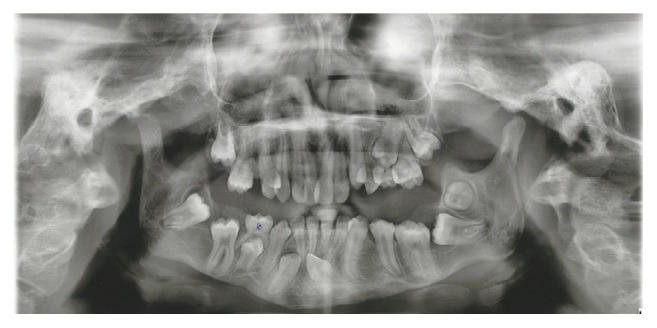
Panoramic radiograph: diverse dental development anomalies, such as maxillary anterior crowding and impacted teeth.

The panoramic radiograph revealed hypoplastic coronoid processes of the mandible, left stylohyoid ligament suggesting ossification, and diverse dental development anomalies, such as maxillary anterior crowding, agenesis of tooth 15, retention of teeth 65 and 85, impacted teeth 33, 37, 43, and 46, and semi-impacted tooth 36 (Figure 9).

**Figure 10:**
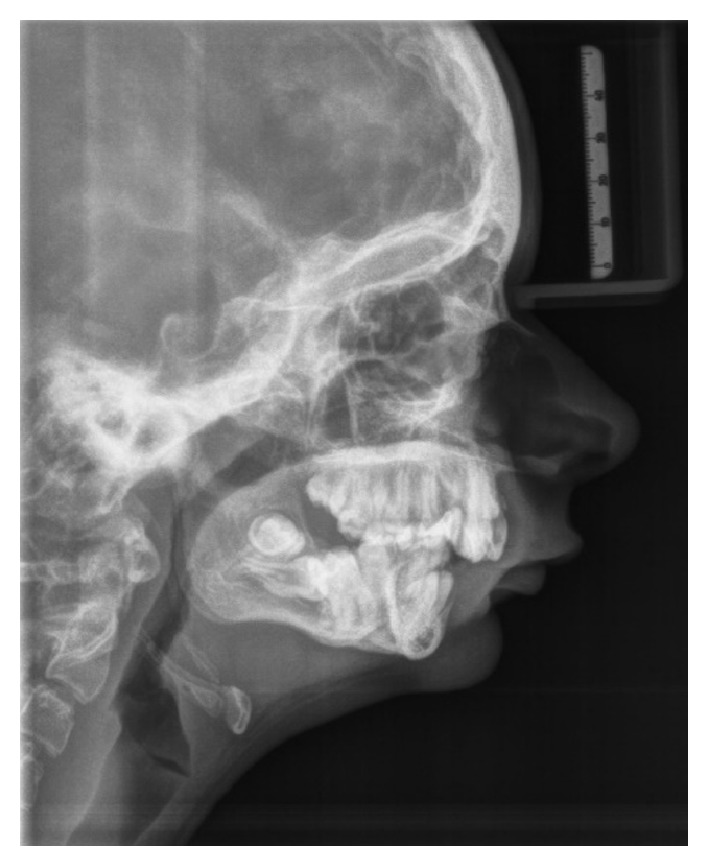
Teleradiography: no proportionality between the effective lengths of the maxilla and mandible.

A teleradiography was requested due to cephalometric analysis, in which we observed that there was no proportionality between the effective lengths of the maxilla and mandible, according to the length of the mandible condyle from the point Co to the point Gn. According to McNamara, for a given size jaw there is a directly proportional mandible size (Figure 10).

### 2.4. Dental Management

The patient was submitted to prophylaxis, advice regarding oral hygiene and topical application of fluoride. As immediate treatment, the primary teeth retained in the arch were extracted due to the fact that these teeth were impeding the normal eruption of the permanent successors.

The patient will continue in medical and dental follow-up with orthodontic accompaniment and there is a probability that she will undergo orthognathic surgery or mandibular distraction after the growth phase. She is also being assisted by a program focused on the prevention of dental caries, periodontal disease, and occlusal problems.

## 3. Discussion

Based on the literature, specific, clinical, and radiological features were found in the patient from the case report: a small face with a prominent front, bulging cheeks, exophthalmia, micrognathia, cranial hyperostosis, dental misalignment, and mandibular hypoplasia. These features were accompanied by short upper limbs, curvature of the long bones, metaphyseal elongation, genu valgum, shortening of the distal phalanges, narrowing of the shoulders and of the pelvis, thinning and irregularities of the ribs, elongation of the vertebrae, and kyphoscoliosis [1, 4, 5, 8–14].

As in the current bibliographic survey, the patient also had hypoplasias of the coronoid process, as well as impaction or absence of the second and third molars and impaction of the canines. They also report the presence of diastemata in the top teeth and slightly conoid lateral incisors, which were not found in this report [23].

Bilateral hypoplasia of the cochlea, leading to loss of hearing, growth hormone deficiency, and hyperelasticity of the skin and joints are anomalies associated with the disease that are reported in the literature review but were not found in the patient [10, 24, 25].

Craniofacial abnormalities and characteristic radiographic findings in our patient suggested the diagnosis of Melnick-Needles Syndrome (MNS). Although the main differential diagnosis of this syndrome includes otopalatodigital syndrome because the syndromes have many clinical and radiological similarities between them, an important differential diagnosis should be done. The appearance of the high vertebral bodies with anterior concavity, bowing of long bones such as radius, ulna, and tibia are commonalities between the two syndromes. The second is characterized by specific malformations and/or disorders of the ears (oto), palate (palate), fingers and toes (digit), skull (cranium), mouth (oro), face (facio), and bones (bone). Some of the features common to both types of OPD syndrome include cleft palate, prominent forehead, broad nose, widely spaced eyes (hypertelorism), inclined downward from the opening between the upper and lower eyelids (palpebral fissures), loss of conductive hearing, short fingers (brachydactyly), an abnormal inside curvature of the fingers (clinodactyly), a recess in the chest in the newborn (pectus excavatum), short stature (dwarfism), and congenital dislocation of the elbow caused by misalignment of the head of the large bone in the forearm (radius). However, the base of the skull sclerosis and cortical irregularities with disorganization of metaphyseal architecture of long bones, typical of MNS and absent in otopalatodigital syndrome, allowed the exclusion of this hypothesis, besides the absence of craniosynostosis, in our patient, which is very characteristic. The similarities of Melnick-Needles Syndrome with otopalatodigital syndrome in relation to craniofacial and skeletal changes allowed the assumption that the two types of skeletal dysplasia are allelic conditions.

The female phenotype for Melnick-Needles Syndrome is variable [8, 10]. Normally, the women affected are short and have delayed motor development, osteoarthritis, hoarse voices, and urethral stenosis, leading to hydronephrosis, in addition to the main abnormalities [8]. There are cases that report that the women affected have difficulty in giving birth normally because of the narrowing of the pelvis [6]. None of these mentioned changes is present in this reported case.

Males born from affected mothers have a similar phenotype to that of affected women, although they generally have more serious complications, such as exophthalmia, sclerocornea, hypertelorism, serious micrognathia, cleft palate, omphalocele, renal hypoplasia, urinary malformations, deformities of the hands and feet, bone dysplasia, and curved long bones [4, 5, 8].

The condition is lethal for boys born to mothers with Melnick-Needles osteodysplasty [4, 5, 8, 11, 13]. The reason for the different clinical manifestations in males born to affected women and those born to normal parents remains unclear. It has been suggested that less affected patients represent new mutations, with an expression that can range from a slighter to a more serious form [9, 13]. The literature contains the case of an 11-year-old boy born to healthy parents, who is considered the oldest patient documented to date [13].

Interpretation of the family data together with radiographic examination of our patient's parents suggests a new mutation. Thus, there does not seem to have been transmission of the gene across different generations, but rather a new event. According to this logic, the chance of a recurrence of the syndrome in the patient's siblings is lower than 1%. Nevertheless, considering the recognized variation in the disturbance's clinical expression, we cannot discount the possibility that the shortening of the terminal phalanx observed in the patient's mother represents a potentially slight expression of the syndrome. In that case, the risk of recurrence in siblings goes up to 50%, with the possibility of a lethal expression in male children.

The possibility that the patient's father was a carrier of the syndrome was discounted based on a review of the literature and on the analysis of radiographs performed during this investigation.

For our patient's children, the risk of recurrence is 50%. For this reason, we recommend genetic counseling when the patient reaches the age of fertility or when the patient wishes it.

Knowledge of this condition becomes relevant, in order to help dental care as well as understand its origin through a genetic aid, so that treatments are covered as possible or according to their limits, thereby relieving the problems associated with the syndrome.

In some cases reported in the literature, the authors described hypoplasia of the coronoid process of the mandible and shortening of the rami. Moreover, the absence of premolars or molars and impaction of second and third molars is something common [14, 16, 17]. Thus, the dental treatment of patients with MNS requires individualized and multidisciplinary attention in order to fix, when possible, dental-skeletal deformities or at least attenuate them. Due to the complexity of this syndrome, in addition to medical professionals, various dental specialties should be involved, such as orthodontists, oral and maxillofacial surgeons, and paediatricians.

## 4. Conclusion

Knowledge of this rare syndrome on the part of dentists is important due to the fact that this condition involves severe abnormalities of the stomatognathic system that exert an impact on the development of the entire face and cause functional and esthetic impairments. Thus, healthcare professionals who are more familiar with this condition could assist in the integrated treatment that MNS requires. By working in an interdisciplinary fashion, dentists, physicians, physiotherapists, psychologists, and geneticists could improve the patient's quality of life, prevent and control complications, and correct irregularities (whenever possible), thereby minimizing the functional, psychological, behavioral, and esthetic impact on patients with Melnick-Needles Syndrome.

## Figures and Tables

**Table 1:** Chart of similarities and differences between the reported syndrome and other syndromes with FLNA gene mutation.

Other syndromes caused by mutations in FLNA gene	Similarities between MNS and other syndromes with FLNA gene mutation	Differences between MNS and other syndromes with FLNA gene mutation
Frontometaphyseal dysplasia	(i) Skeletal dysplasia	(i) Supraorbital hyperostosis (ii) Irregular cortex in long bones (iii) Deafness (iv) More severe skeletal and facial manifestations than OPD1 (v) Metacarpophalangeal and interphalangeal joint defects

Otopalatodigital dysplasia type I	(i) Deformities on the tips of fingers and toes (ii) Abnormal palate	(i) Among all, the phenotype is milder in men (ii) Supraorbital hyperostosis (iii) Deafness (iv) Slightly reduced stature

Otopalatodigital dysplasia type II	(i) Abnormal palate	(i) More severe than type I (ii) Facial dimorphism (iii) Hypoplasia of thorax (iv) More accentuated anomalies in fingers and toes (v) Hydrocephalus, obstructive uropathy, heart defects, and omphalocele (vi) Most die in prenatal period or childhood

**Table 2:** Chart of the main syndromes and characteristics that should be considered in differential diagnosis with Melnick-Needles Syndrome.

Syndromes with similar characteristics	Similarities between MNS and other syndromes	Differences between MNS and other syndromes
Pierre Robin Sequence	(i) Craniofacial anomalies (ii) Micrognathia	(i) Glossoptosis (ii) Hypoplasia of the mandible (iii) Cleft palate

Treacher Collins Syndrome	(i) Craniofacial anomalies (ii) Micrognathia	(i) Antimongoloid inclination of eyelid fissures (ii) Coloboma of the lower eyelid (iii) Zygomatic, maxillary, and mandibular hypoplasia (iv) Complete or partial absence of eyelashes on lower eyelids (v) Malformations of the outer ears (vi) Hair follicles between ear and angle of the mouth (vii) Cleft palate (in some cases)

Frank-Ter Haar Syndrome	(i) Multiple skeletal anomalies (ii) Prominent brow (iii) Micrognathia (iv) Prominent eyes	(i) Autosomal recessive (ii) Heart malformations (iii) Prominent coccyx (iv) Delayed development (v) Congenital glaucoma may be associated (vi) Hypertelorism

Shprintzen-Goldberg Syndrome	(i) Exophthalmia (ii) Prominent brow (iii) Narrow palate (iv) Scoliosis	(i) Craniosynostosis (premature closure of cranial sutures) (ii) Extremely rare (iii) Dolichocephaly (iv) Hypertelorism (v) Mandibular hypoplasia (vi) Oblique eyes, low position of ears (vii) Long, thin fingers

Crouzon Syndrome	(i) Exophthalmia	(i) Craniosynostosis (ii) Craniofacial anomalies: hypertelorism, strabismus, “parrot-beak” nose, short lower lip, hypoplasia of maxilla, and mandibular prognathism

## Abbreviations

MNS: Melnick-Needles Syndrome
OMIM: Online Mendelian Inheritance in Man
FLNA: Filamin A, alpha.
